# Supplementary material for: A Randomized, Double-Blind, Placebo-Controlled, Multicenter Study to Evaluate the Safety and Efficacy of ThymoQuinone Formula (TQF) for Treating Outpatient SARS-CoV-2
Source: Pathogens. 2022 May 7;11(5):551. doi: 10.3390/pathogens11050551 (PMC9144779; doi:10.3390/pathogens11050551)
Supplement: Supplementary file 1 [file pathogens-11-00551-s001.zip › pathogens-1717026-supplementary.pdf]

A Randomized, Double-Blind, Placebo-Controlled, Multicenter Study to Evaluate the Safety and Efficacy of TQ Formula for Treating outpatient SARS-CoV-2

Supplementary

Contents

SUPPLEMENTARY FIGURES

Supplementary Figure S1: 3-dimensional modeling of viral spike protein, angiotensin-converting enzyme 2 (ACE2) receptor with thymoquinone.

Supplementary Figure S2: Inhibition of SARS-CoV-2 pseudoviruses infection by TQ Formula and TQ.

Supplementary Figure S3: Inhibition of SARS-CoV-2 pseudoviruses entry by TQ Formula and TQ.

Supplementary Figure S4: Model-based change of throat, gastrointestinal, chest/respiratory body/systemic symptom burden by study arm.

SUPPLEMENTARY TABLES

Supplemental Tables S1: The Molecules and Modeling Docking Process.

Supplementary Table S2: Flow Cytometry Comparison of T lymphocytes at Day-14.

SUPPLEMENTARY FIGURES

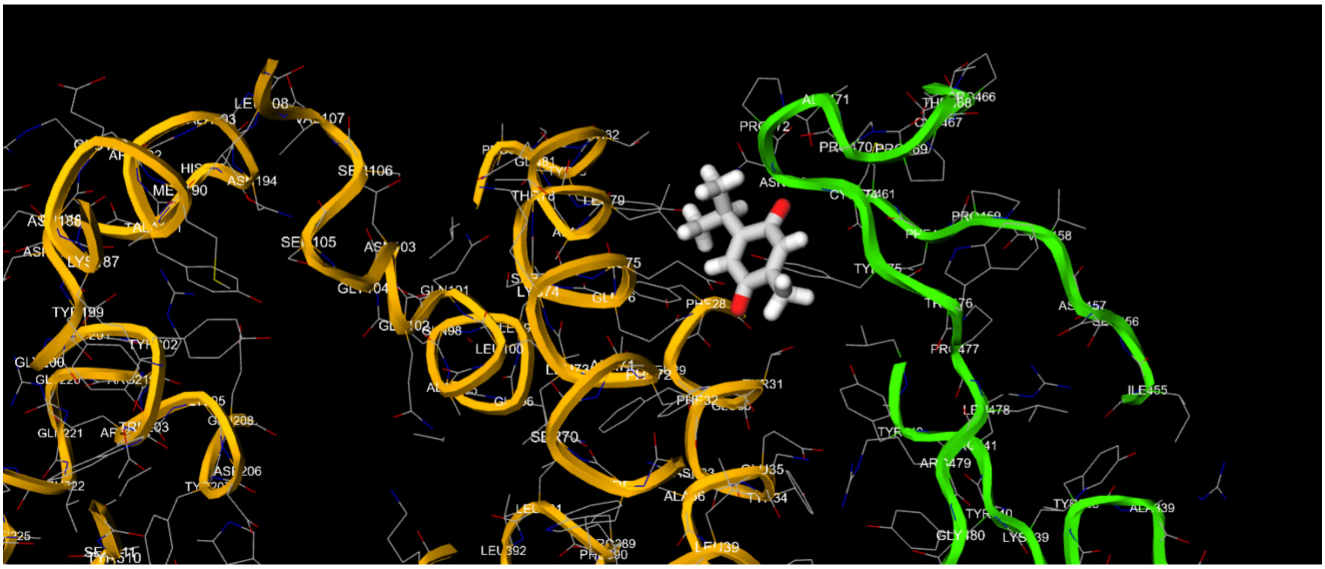

Figure S1. Three dimensional modeling of viral spike protein (green), angiotensin-converting enzyme 2 (ACE2) receptor (yellow) with thymoquinone (white).

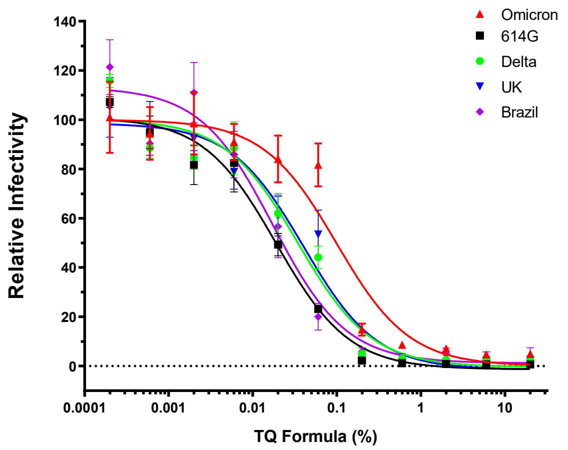

	Omicron	614G	Delta	UK	Brazil
Bottom	0.03621	-1.319	-0.4204	-1.478	1.176
Top	100.0	101.0	100.2	98.59	113.2
LogIC50	-1.006	-1.721	-1.460	-1.396	-1.758
IC50	0.09862	0.01900	0.03467	0.04021	0.01745
Span	99.97	102.4	100.6	100.1	112.0

(**A**)

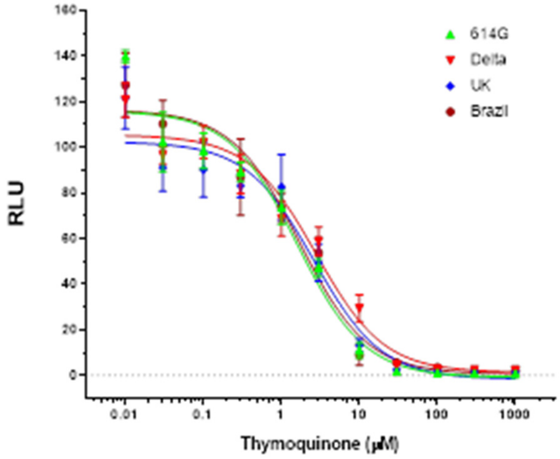

	614G	Delta	UK	Brazil
Bottom	-0.8732	1.245	-1.634	0.5306
Top	115.9	105.4	102.4	116.4
LogIC50	0.2017	0.4431	0.4154	0.2301
IC50	1.591	2.774	2.603	1.699
Span	116.8	104.2	104.0	115.9

(**B**)

Figure S2. Inhibition of SARS-CoV-2 pseudoviruses infection by TQ Formula and TQ. (**A**) Serial dilutions of TQ Formula were tested for inhibition against an MLV-based pseudotyped virus using four different SARS-CoV-2 variant spike protein constructs (Omicron, 614D, Delta, UK, Brazil), in the infection of HEK293-ACE2 cells. TQ Formula showed inhibitory effect on all four SARS-CoV-2 variants with an IC50 value range between 0.01% to 0.04%. (**B**) Serial dilutions of TQ were tested for inhibition against an MLV-based pseudotyped virus using four different SARS-CoV-2 variant spike protein constructs (614D, Delta, UK, Brazil), in the infection of HEK293-ACE2 cells. Thymoquinone showed inhibitory effect on all four SARS-CoV-2 variants with an IC50 value range between 1-3 μM. X-axis: compound concentration. Y-axis, relative luminescence unit (RLU), reflecting the luciferase activity and the viral infectivity. The experiments were repeated three times with independent samples giving similar results. For all panels, data points shown are mean and s.d. for n = 3 technical replicates. IC50 values derived from curve fitting are listed in the Table below each Graph.

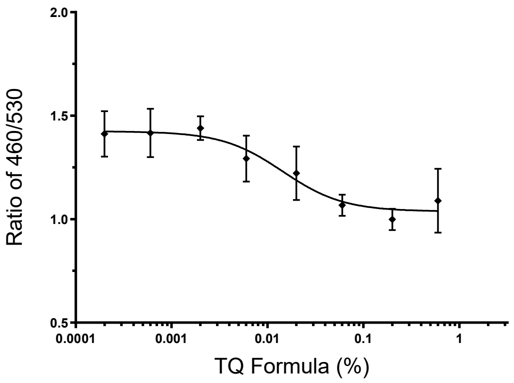

	TQ Formula
Bottom	1.037
Top	1.425
LogEC50	-1.842
HillSlope	-1.370
EC50	0.01439
Span	0.3872

(**A**)

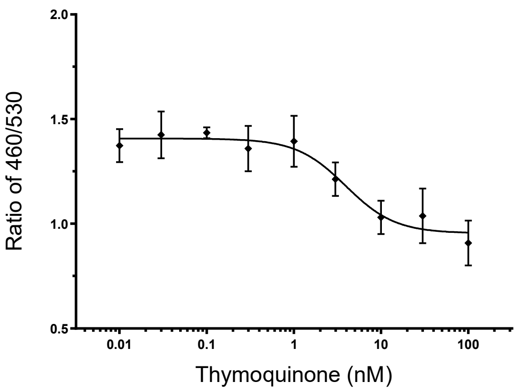

	Thymoquinone (nM)
Bottom	0.9546
Top	1.407
LogEC50	0.5949
HillSlope	-1.532
EC50	3.934
Span	0.4523

(**B**)

Figure S3. Inhibition of SARS-CoV-2 pseudoviruses entry by TQ Formula and TQ. Serial dilutions of TQ Formula and TQ were tested for inhibition against an MLV-based pseudotyped virus in the entry of HEK293-ACE2 cells. The beta lactamase was fused to the N-terminus of MLV-gag-pol protein (bLac-gag-pol). The SARS-CoV-2 variant spike protein construct is 614D. X-axis: compound concentration. Y-axis, ration of 460nm/530nm, reflecting the beta lactamase activity and the viral entry into cells. The experiments were repeated three times with independent samples giving similar results. For all panels, data points shown are mean and s.d. for n = 3 technical replicates. IC50 values derived from curve fitting are listed in the Table below each Graph. Both TQ Formula (**A**) and TQ (**B**) can inhibit SARS-CoV-2 614D variant entry into ACE2-expressing HEK293 cells.

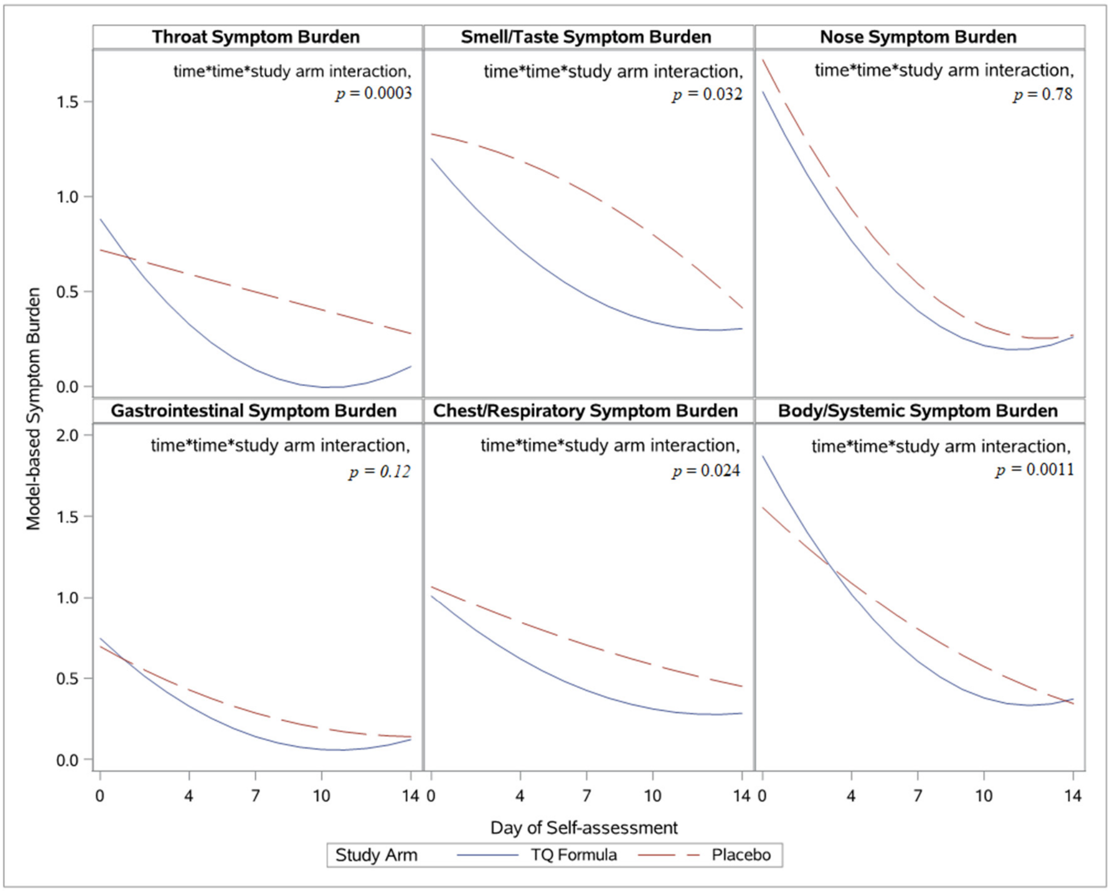

Figure S4. Model-based change of throat, smell/taste, nose, gastrointestinal, chest/respiratory, and body/systemic symptom burden by study arm. Shown are the Model-based change of throat, gastrointestinal, chest/respiratory body/systemic symptom burden for TQF (blue) and placebo (red) arms.

SUPPLEMENTARY TABLES

Table S1. The Molecules and Modeling Docking Process.

To evaluate the ability of the major key components of TQ Formula (thymoquinone, TQ), we docked TQ at the protein target where viral spike protein interface with ACE2 receptor. The details of the molecules (Table S1A) and modeling docking process details (Table S1B) are summarized in the tables below:

A- Molecules:

Molecule Name	Nature	Details (source file/Canonical SMILES)
3SCK	Protein complex: Angiotensin-converting enzyme 2 chimera (chains A,B); AND Spike glycoprotein CHANS C and D	PDB file @ https://www.rcsb.org/structure/3SCK
Thymoquinone	Small Molecule	CC1=CC(=O)C(=CC1=O)C(C)C

B- Modeling details:

Item	Details
Software for docking	https://mcule.com/apps/1-click-docking/
Software for interface analysis	https://www.ebi.ac.uk/pdbe/pisa/
Protein target for docking	Geometric pocket center at (43.53, −4.71, 125.30) with max radius 15.71 Angstrom

Table S2. Flow Cytometry Comparison of T lymphocytes at Day-14.

Flow Cytometry Markers			Marker Value							
			N	Min	Q1	Median	Mean	SD	Q3	Max
	p-value	Placebo	20	0.00	4.00	6.50	8.00	6.17	10.00	22.00
% CD45RA+CCR7+ (% CD4 T) (% of CD4 T cell)	0.025*	Blackseed Oil	25	11.00	28.00	37.00	37.68	14.14	44.00	68.00
		Placebo	20	10.00	17.00	24.50	27.40	12.08	38.00	52.00
% CD45RA+CCR7+ (% CD8 T) (% of CD8 T cell)	0.038*	Blackseed Oil	25	12.00	28.00	43.00	42.76	20.55	55.00	83.00
		Placebo	20	10.00	19.50	26.50	29.80	13.62	41.00	57.00
% CD45RA+CCR7- (% CD8 T) (% of CD8 T cell)	0.20	Blackseed Oil	25	0.00	7.00	20.00	21.40	17.42	31.00	59.00
		Placebo	20	1.00	17.50	24.00	26.20	13.08	34.50	49.00
% CD45RA- CCR7+ (% CD8 T) (% of CD8 T cell)	0.32	Blackseed Oil	25	1.00	8.00	13.00	16.12	12.13	18.00	48.00
		Placebo	20	4.00	12.00	15.00	17.25	8.75	22.00	41.00
% CD45RA-CCR7+ (% CD4 T) (% of CD4 T cell)	0.094	Blackseed Oil	25	19.00	38.00	45.00	46.16	15.42	52.00	87.00
		Placebo	20	33.00	45.50	51.00	51.60	10.29	58.00	77.00
% CD45RA-CCR7- (% CD4 T) (% of CD4 T cell)	0.16	Blackseed Oil	25	0.00	8.00	14.00	13.68	9.25	17.00	40.00
		Placebo	20	0.00	11.00	16.50	16.80	8.04	21.00	33.00
% CD45RA-CCR7- (% CD8 T) (% of CD8 T cell)	0.081	Blackseed Oil	25	0.00	7.00	18.00	17.88	11.45	28.00	34.00
		Placebo	20	0.00	15.00	26.00	25.00	12.69	35.00	44.00
% CD8+ CD4+ T (% CD3 T) (% of T cell)	0.95	Blackseed Oil	25	0.00	0.00	0.00	0.12	0.33	0.00	1.00
		Placebo	20	0.00	0.00	0.00	0.40	1.39	0.00	6.00
% CXCR3+ (% CD4 T) (% of CD4 T cell)	0.26	Blackseed Oil	25	23.00	38.00	42.00	48.28	20.53	51.00	99.00
		Placebo	20	28.00	41.00	47.50	48.25	11.76	57.50	65.00
% CXCR3+ (% CD8 T) (% of CD8 T cell)	0.69	Blackseed Oil	25	41.00	58.00	69.00	69.04	15.79	78.00	99.00
		Placebo	20	45.00	57.00	66.50	66.15	13.12	75.50	90.00

Abbreviations: N, number; Q1, first quadrantile; Q3, third quadrantile; SD, standard deviation; min, minimum; max, maximum

* Statistically significant
